# The Care of the Dentists’ Hands

**Published:** 1881-12

**Authors:** T. F. Chapin

**Affiliations:** Dental Office and Laboratory


### THE CARE OF THE DENTIST'S HANDS.

For many years past I have been very much annoyed, and, sometimes, almost incapacitated for work as soon as the winter months would set in, by the condition of my hands, the result of sores from the chapping of the skin and cracking of the flesh, brought about by the frequent washing of the hands in going from one patient to another, etc.

The remedy is simple, but its application must be constant and unremitting.

To one pound of pure glycerine I add two ounces of rose water, shaking these together until well mixed. On my wash-stand, in both office and laboratory, I keep a small toy pitcher, that will hold about an ounce of the above mixture, and *every time I wash my hands, before wiping them,* I pour into the palm of one hand a few drops of this glycerine, rubbing it over both hands, as one would do in the act of soaping them, and then wipe them dry.

I would impress, however, on those who wish to try it, that it must be done *unremittingly.* If the hands are washed fifty or one hundred times a day, fifty or one hundred applications of the remedy must be made.

T. F. Chapin, in the *Dental Office and Laboratory.*

---

Canon Rawstorne says, in a late sermon, "It is patent to everybody that those professions which are doing a real work tolerate but little ceremonial. There was a time when the doctor of medicine had as many paraphernalia as a divine; and had a regular routine with all cases, like that ridiculed by Moliere and Le Sage, curing, or perhaps killing, but always acting *selon les regles, secumdem artem.* All that is quite gone. A doctor who tried to envelop himself in ceremony should be set down at once as a charlatan; as he would no doubt be. All medical subjects are submitted to the freest investigation; everything is reduced to bare scientific truth, and mere reality."

